# ParasitoBank dataset for diagnosing intestinal parasitism: Helminths and protozoa in coprological samples

**DOI:** 10.1016/j.dib.2025.111279

**Published:** 2025-01-08

**Authors:** Jader Alejandro Muñoz Galindez, Luis Reinel Vásquez Arteaga, Rubiel Vargas Cañas

**Affiliations:** a Sistemas dinámicos, instrumentación y control (SIDICO), Departamento de física, Universidad del Cauca, Colombia; b Centro de estudios en microbiología y parasitología (CEMPA), Departamento de medicina interna, Universidad del Cauca, Colombia

**Keywords:** Antiparasitic, Health, Optical microscopy, Tropical diseases, Treatment

## Abstract

Intestinal parasitism is an infection that affects people worldwide, with populations in developing countries being at a higher risk of acquiring it. This infection is contracted for various reasons, mainly related to poor sanitary conditions and inadequate food practices, leading to multiple health issues such as malnutrition, intestinal obstructions, epilepsy, and others. Identifying parasitic species is essential for establishing appropriate antiparasitic therapy, which in turn helps reduce the risk of associated morbidities. For this reason, a dataset named “ParasitoBank” was created, containing 779 images of the visual field of fresh stool samples analysed under a microscope using the serial coprological technique. These images were acquired using a Motorola G84 mobile phone, and a data-labeling process resulted in a total of 1,620 intestinal parasites, with a particular focus on intestinal protozoa. The images have an approximate aspect ratio of 1:1 with a resolution of 2100 × 2100. Label information and some metadata for the images have been included in a JSON file following the “Common Objects in Context” (COCO) format. Finally, the entire dataset and label content have been arranged in a compressed file. The presented information facilitates the use of the data for various studies, spanning education and artificial intelligence development.

Specifications Table

**Table:** 

Subject	Computer Vision and Pattern Recognition
Specific subject area	Recognition of Intestinal Parasitism for Supporting Parasitological Diagnosis.
Type of data	Images in JPG format with diverse content of intestinal parasites
Data collection	This dataset contains 779 images of the visual field of fresh stool samples analyzed under a microscope. The images were acquired using a Motorola G84 mobile phone, with resolutions of 3072 × 3072 pixels and 3072 × 4080 pixels. The parasitic content was labeled by delineating the regions, resulting in a total of 1620 intestinal parasites, with a particular focus on intestinal protozoa. Through image processing, regions of no interest and noise that did not provide relevant information within the visual field were removed, achieving images with an approximate aspect ratio of 1:1 and a resolution of 2100 × 2100 pixels. The labeling information was organized in a JSON file, which was packaged together with the folder containing the images in a compressed file.
Data source location	Institution: Universidad del Cauca, City/Region: Popayán-Cauca, Country: Colombia
Data accessibility	Repository name: Mendeley Data Data identification number: 10.17632/725hpzpwzf.1 Direct URL to data: https://data.mendeley.com/datasets/725hpzpwzf/1
Related research article	

## 1 Value of the Data

• This information showcases a wide variability of intestinal parasites, consisting of 1531 images of protozoa and 89 images of helminths, all captured within the visual field of mounts prepared using the serial coprological technique.
• The dataset can be highly useful for researchers in fields such as Technology, Bacteriology, Microbiology, Parasitology, Biology, and Medicine, who are interested in evaluating and teaching parasitological diagnosis.
• It offers the opportunity to visualize the life cycles of intestinal parasites in mounts, as observed through a microscope, providing a realistic representation of the use of optical microscopy.
• It provides the opportunity to develop artificial intelligence systems specialized in the identification of intestinal parasites, aimed at creating advanced applications that serve as support tools for diagnosis in clinical laboratories.
• This dataset would enable researchers to deepen their understanding of the forms and life cycles of various parasitic species, serving as a valuable complement for studies requiring detailed representations of parasites.

## 2 Background

Neglected tropical diseases primarily affect developing countries in tropical and subtropical regions. Among these diseases are those associated with intestinal parasitism, caused by helminths and intestinal protozoa [1]. The main causes of parasitic infections include inadequate water purification, undercooked food, improper waste management, lack of personal hygiene, and the educational level of individuals [2]. To ensure proper treatment, determining the parasitic species involved is crucial for administering antiparasitic therapy [3]. In this context, artificial intelligence has been involved in the recognition of parasitic species from images at different stages of their life cycle. Although research has mainly focused on the detection of helminths [4,5], thanks to the increased availability of resources such as the ChullaEgg11 repository [6], protozoan identification remains a challenge due to their small size, which in some cases is confused with food remnants, and the limited number of studies [7]. Additionally, research often uses high-resolution microscope cameras, which offer a considerable level of detail but do not reflect the real diagnostic conditions present in many clinical laboratories. This combination of factors has created a significant gap, especially in providing practical solutions for laboratories in remote areas, where resources are limited for patient care.

## 3 Data Description

**Fig. 1:**
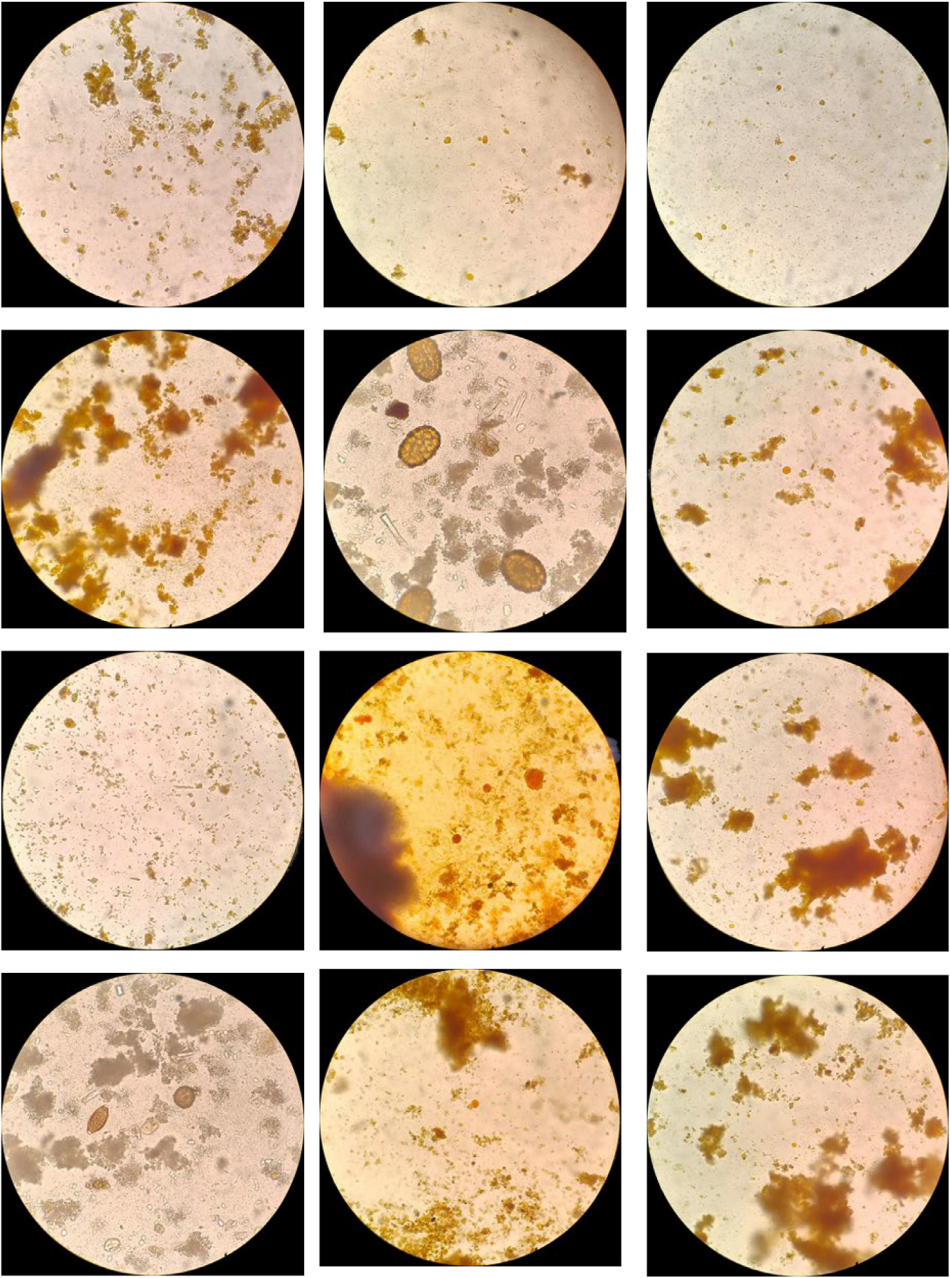
Format of the images in the dataset.

**Fig. 2:**
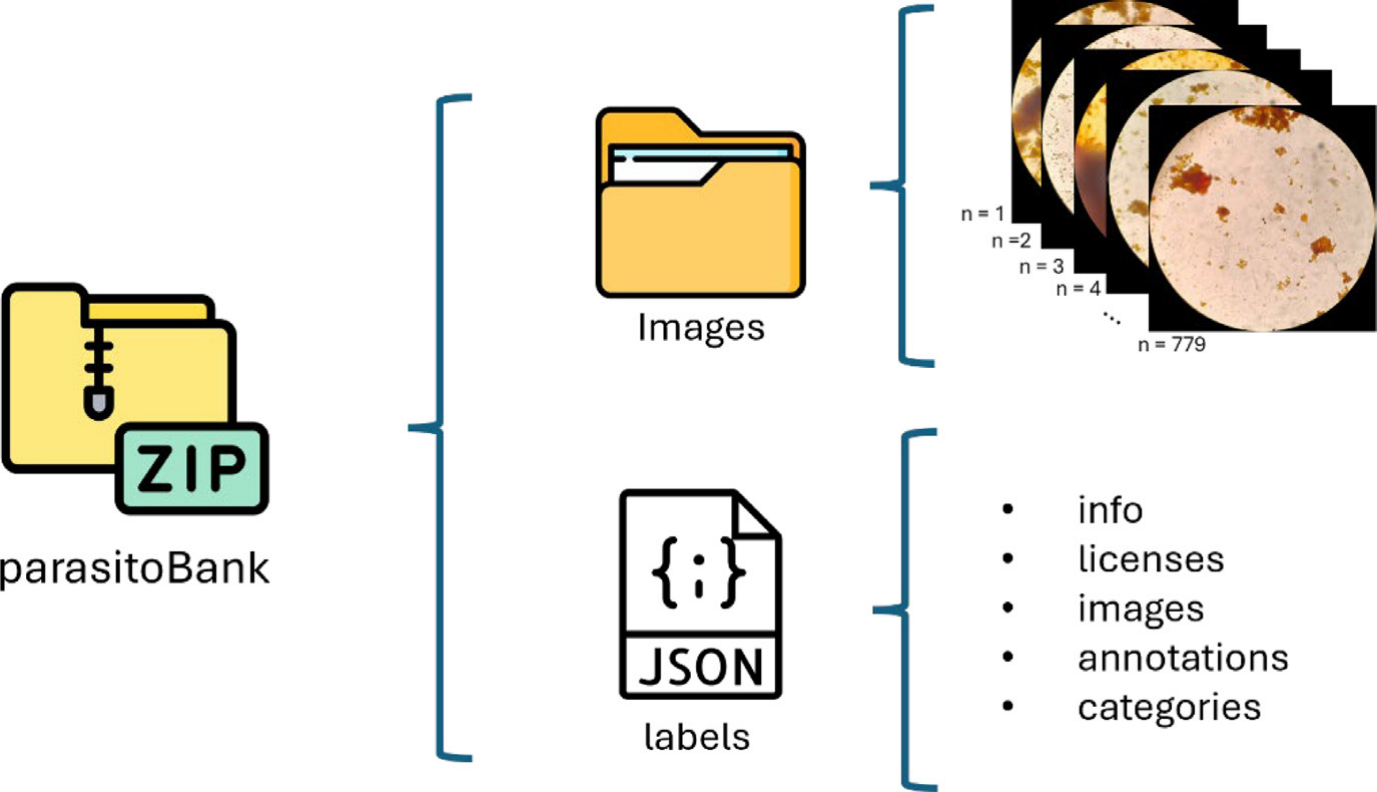
Contents of the dataset ParasitoBank.

The dataset consists of 779 images in JPG format obtained through the eyepiece of a microscope, with a primary focus on species of intestinal protozoa. These images have been collected from a series of fresh human stool samples examined using the serial coprological method. Various reagents, such as Lugol and saline solution, were used in processing the samples, providing a varied background color across the different samples. To reduce the number of irrelevant areas, image processing was performed to isolate only the ocular field (Fig. 1). Each image file has been renamed following a specific template: “parasiteField” followed by a number ranging from zero to the total number of images. This naming convention facilitates the referencing and organization of the data for quick utilization. Additionally, using the “Common Objects in Context” (COCO) [8] format as a reference, a JSON file is attached, containing the positions of the parasites, categories, and other metadata, alongside a folder with all the images (Fig. 2), like what has been done in the “Chula-ParasiteEgg-11” repository [6]. In total, there are labels for 1620 intestinal parasites, distributed across 1531 images of protozoa and 89 helminths. Table 1 presents the total, detailing the number of species identified throughout the dataset.

**Table 1:** Number of intestinal parasites in the dataset ParasitoBank.

Type	Species	Amount
Protozoa	*Blastocystis*	403
	*Chilomastix mesnili*	193
	*Intestinal coccidia.*	44
	*Entamoeba complex*	10
	*Endolimax nana*	326
	*Entamoeba coli*	149
	*Entamoeba hartmanni*	135
	*Giardia duodenalis*	209
	*Iodamoeba bütschlii*	62
Helminths	*Ascaris lumbricoides*	57
	*Strongyloides*	17
	*Taenia spp*	5
	*Trichuris trichiura*	10
	Total	1620

## 4 Experimental Design, Materials and Methods

### 4.1 Data acquisition

**Fig. 3:**
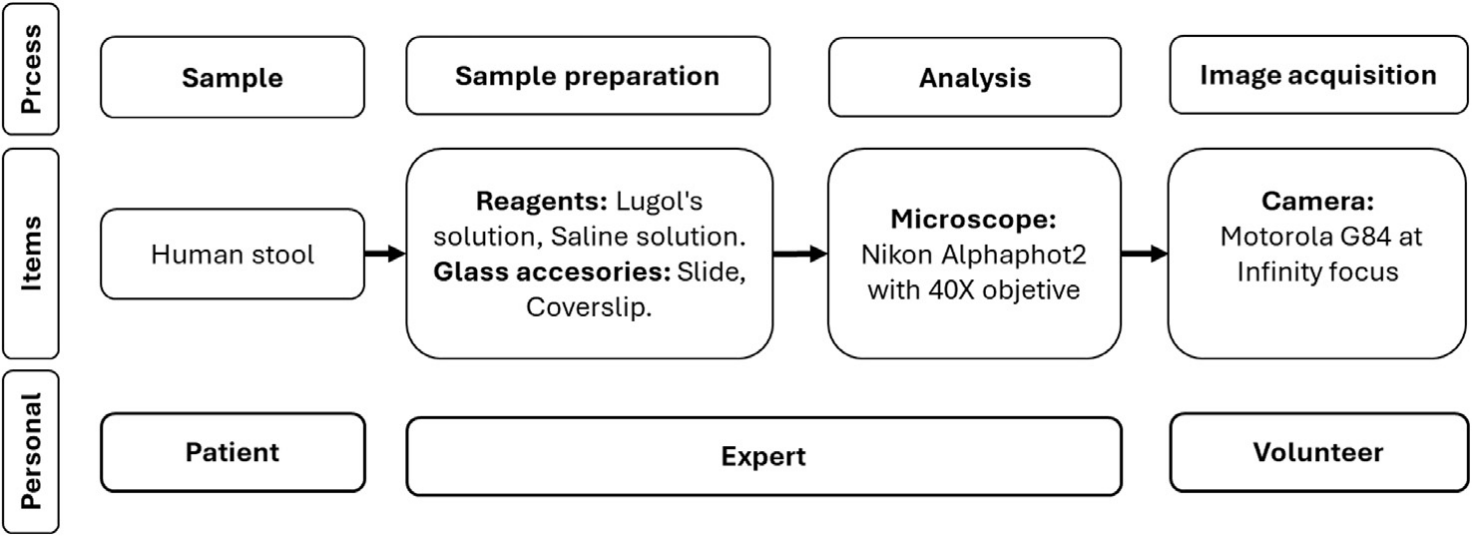
Image acquisition procedure.

The procedure for acquiring the information included the use of fresh human stool samples taken to the CEMPA research group laboratory at the Faculty of Health of the University of Cauca, Colombia. These samples came from various prevalence studies conducted in the department of Cauca. The preparation of the samples and their analysis were carried out by a specialist in parasitology. Using a serial coprological procedure that utilized reagents such as saline solution and Lugol, the fecal sample was processed to detect different intestinal parasites. This exercise was performed with a NIKON Alphaphot-2 microscope at a magnification of 40X, ideal for detecting most intestinal parasites. Additionally, a volunteer present during the clinical procedure used the camera of a Motorola G84 phone in professional mode, focusing to infinity and with other parameters set to automatic (white balance, exposure time, ISO, and exposure), at resolutions of 3072 × 3072 pixels and 3072 × 4080 pixels with aspect ratios of 1:1 and 3:4, respectively, to capture images in JPG format through the eyepiece of the microscope when the professional detected a parasite in the visual field. Subsequently, the regions where the parasite was located were marked and assigned a name (Fig. 3).

### 4.2 Data processing

**Fig. 4:**
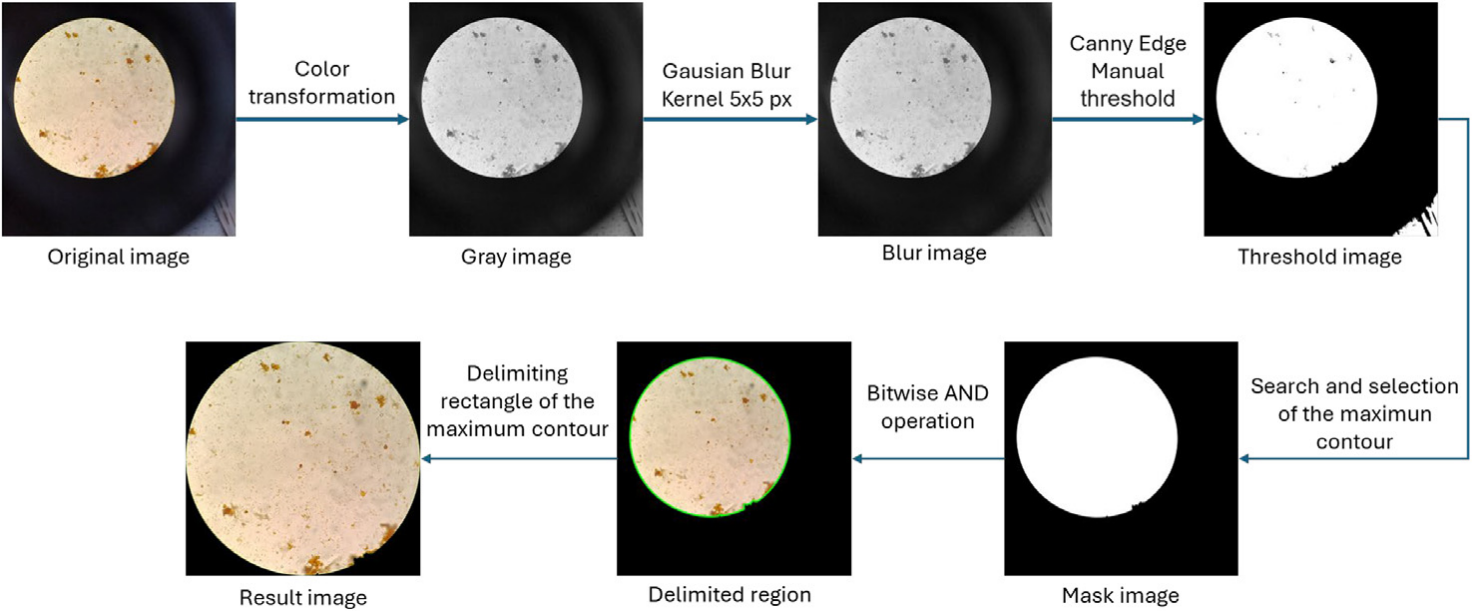
Process of selecting region of interest.

With the aim of optimizing image analysis, irrelevant areas outside the region of interest were removed to reduce the amount of worthless information and improve its utility. This process was carried out using an algorithm developed in Python with the open-source library OpenCV, which applies a series of transformations to identify the largest contour in the image. The procedure includes: conversion to grayscale, application of a Gaussian filter with a 5 × 5 pixel kernel, threshold detection and application, segmentation, logical operations to combine the detected elements, and finally, detection of the rectangular region of the contour (Fig. 4), resulting in an image with an approximate aspect ratio of 1:1 and a resolution of 2100 × 2100 pixels. Each image was processed in this way to optimize contour detection; however, due to variations in lighting, it is not always possible to delineate a perfectly circular contour, especially in regions with poorly defined edges that do not contain intestinal parasites, which can affect the proportions and aspect ratio of the resulting figures.

### 4.3 Labeling and storage process

The labeling procedure was carried out using the software labelImg [9], which allows users to select a region of interest and assign it a name. In this process, efforts were made to maintain an independent labelling style for each species within the same image, aiming to achieve a relationship like that found in a clinical laboratory, where multiple species can coexist within the same visual field.

**Fig. 5:**
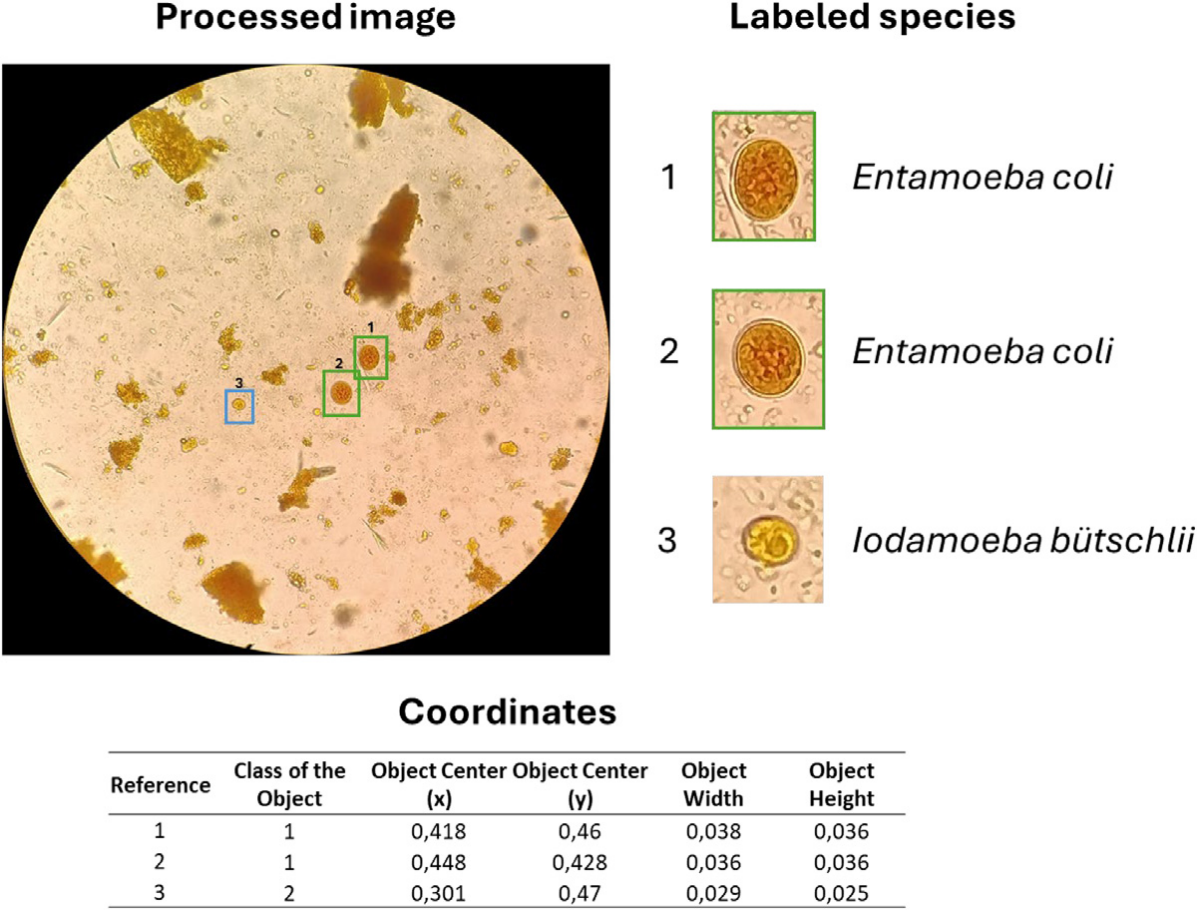
Image labeling process.

The software settings were adjusted to generate labels in YOLO format, where the layout includes an index referencing the parasitic species index, the center of the object (x), the center of the object (y), width, and height. This process generates a text file with the name of the image containing all this information. The previously informally labeled content provided by the expert was considered, and the species content was distributed in the software to label all the images. The goal was not only to cover the object of interest but also to provide a few pixels of background context to obtain more information about the parasite's arrangement (Fig. 5). At this stage, images with better resolution were selected to allow for more effective differentiation of the intestinal parasites present.

On the other hand, to avoid data accumulation that could complicate information management, all text elements were grouped into a JSON file, following the structure of datasets in COCO format. This file summarizes all the information and presents a data structure divided into five sections:

• Info: Provide information about the version, year of creation, description, and creators of the dataset.
• Licenses: Indicate the use of the Attribution 4.0 International open license.
• Images: Store an identifier, the name of the image, and its size.
• Annotations: Include an identifier, the image identifier, the category index, and the coordinates of the bounding box and the area of the region.
• Categories: It contains an identifier and the name of each category.

It enables independent control of each region of interest within the complete image. Each region can be analysed separately, making it easier to extract metadata and location information. Additionally, it enables a detailed examination of the intestinal parasites present in each region, providing a more focused visualization of the parasites. This process is intended for the detection of objects similar to that observed in the “Chula-ParasiteEgg-11” repository [6] and allows for cleaner content management. Finally, the entire dataset has been packaged into a compressed “zip” file that includes a folder named “images.” This arrangement allows for all the content to be gathered in one place for easier handling.

## Limitations

Limitations may arise due to the possible movement of intestinal parasites during image acquisition, which could lead to distortions in their actual shapes. Additionally, the focus of the visual field when capturing the image may not be optimal, making it difficult to view certain areas.

## Ethics Statement

In the context of this study, it is important to note that during the acquisition of images under the microscope, no personal information that could identify the donors is collected. Since the samples are used solely for academic purposes, it is deemed unnecessary to request informed consent for the use of the obtained information. This approach ensures confidentiality and respect for participants, aligning with the ethical principles of scientific research.

## Credit Author Statement

**Jader Muñoz:** Data curation, Conceptualization, Methodology, Software, Writing – original draft, Writing – review & editing; **Luis Vásquez:** Data curation, Conceptualization, Methodology, Writing – review & editing; **Rubiel Vargas**: Conceptualization, Methodology, Writing – review & editing.

## Data Availability

Mendeley DataParasitoBank: Dataset of helminths and protozoa in coprology samples (Original data).
